# Promoter Demethylation Upregulates *STEAP1* Gene Expression in Human Prostate Cancer: In Vitro and In Silico Analysis

**DOI:** 10.3390/life11111251

**Published:** 2021-11-17

**Authors:** Sandra M. Rocha, Inês Sousa, Inês M. Gomes, Patrícia Arinto, Pedro Costa-Pinheiro, Eduarda Coutinho, Cecília R. Santos, Carmen Jerónimo, Manuel C. Lemos, Luís A. Passarinha, Sílvia Socorro, Cláudio J. Maia

**Affiliations:** 1 CICS-UBI-Health Sciences Research Center, Universidade da Beira Interior, 6201-506 Covilhã, Portugal; sandra.rocha@ubi.pt (S.M.R.); inesmmsousa@ua.pt (I.S.); inesgomes@fcsaude.ubi.pt (I.M.G.); patricia_arinto@hotmail.com (P.A.); ecoutinho@fcsaude.ubi.pt (E.C.); csantos@fcsaude.ubi.pt (C.R.S.); mclemos@fcsaude.ubi.pt (M.C.L.); lpassarinha@fcsaude.ubi.pt (L.A.P.); ssocorro@fcsaude.ubi.pt (S.S.); 2 Department of Medical Sciences, Institute of Biomedicine—iBiMED, Universidade de Aveiro, 3810-193 Aveiro, Portugal; 3 Cancer Biology and Epigenetics Group, IPO Porto Research Center (CI-IPOP), Portuguese Oncology Institute of Porto (IPO Porto), 4200-072 Porto, Portugal; pedro.dacostapinheiro@pennmedicine.upenn.edu (P.C.-P.); carmenjeronimo@ipoporto.min-saude.pt (C.J.); 4 Department of Pathology and Molecular Immunology, School of Medicine and Biomedical Sciences, Universidade do Porto (ICBAS-UP), 4050-513 Porto, Portugal; 5 C4-UBI, Cloud Computing Competence Center, Universidade da Beira Interior, 6200-501 Covilhã, Portugal; 6 Associate Laboratory i4HB-Institute for Health and Bioeconomy, NOVA School of Science and Technology, Universidade NOVA de Lisboa, 2819-516 Caparica, Portugal; 7 UCIBIO-Applied Molecular Biosciences Unit, Department of Chemistry, NOVA School of Science and Technology, Universidade NOVA de Lisboa, 2819-516 Caparica, Portugal; 8 Laboratório de Fármaco-Toxicologia-UBIMedical, Universidade da Beira Interior, 6201-284 Covilhã, Portugal

**Keywords:** prostate cancer, *STEAP1*, DNA methylation, histone deacetylation, bioinformatics

## Abstract

The Six Transmembrane Epithelial Antigen of the Prostate (*STEAP1*) is an oncogene overexpressed in several human tumors, particularly in prostate cancer (PCa). However, the mechanisms involved in its overexpression remain unknown. It is well known that epigenetic modifications may result in abnormal gene expression patterns, contributing to tumor initiation and progression. Therefore, this study aimed to analyze the methylation pattern of the *STEAP1* gene in PCa versus non-neoplastic cells. Bisulfite amplicon sequencing of the CpG island at the *STEAP1* gene promoter showed a higher methylation level in non-neoplastic PNT1A prostate cells than in human PCa samples. Bioinformatic analysis of the GEO datasets also showed the *STEAP1* gene promoter as being demethylated in human PCa, and a negative association with *STEAP1* mRNA expression was observed. These results are supported by the treatment of non-neoplastic PNT1A cells with DNMT and HDAC inhibitors, which induced a significant increase in *STEAP1* mRNA expression. In addition, the involvement of HDAC in the regulation of *STEAP1* mRNA expression was corroborated by a negative association between *STEAP1* mRNA expression and *HDAC4,5,7* and *9* in human PCa. In conclusion, our work indicates that *STEAP1* overexpression in PCa can be driven by the hypomethylation of *STEAP1* gene promoter.

## 1. Introduction

Prostate cancer (PCa) is the second most common cancer worldwide, and it is the sixth leading cause of cancer death globally [1]. Prostatic carcinogenesis arises from precursor preneoplastic lesions that have the distinct molecular characteristics of normal prostate cells, and may give rise to localized cancer and eventually acquire the potential of invasion and metastasis [2]. The molecular alterations underpinning these cellular events include mutations, gene deletions and amplifications, chromosomal rearrangements and epigenetic modifications in key genes controlling cell fate [3,4,5]. The main epigenetic mechanisms are DNA methylation and histone modifications, the main function of which is to ensure the proper regulation of gene expression by changing the chromatin structure [6]. DNA methylation is catalyzed by the family of enzymes known as DNA methyltransferases (DNMTs) and by histone modifications, including mainly histone acetylation, which is regulated by two groups of enzymes exerting opposite effects, histone acetyltransferases (HATs) and histone deacetylases (HDACs) [5,6].

In tumor cells, hypermethylation is observed in promoters of specific genes, particularly tumor suppressor genes, and a global hypomethylation contributes to genomic instability and the activation of oncogenes [7]. The disruption of epigenetic mechanisms may conduct the deregulation of gene expression, leading to tumor development and progression [6].

The Six Transmembrane Epithelial Antigen of the Prostate 1 (*STEAP1*) gene was identified as overexpressed in PCa compared to non-malignant tissues [8]. The STEAP1 protein is mainly located in the plasma membrane of epithelial cells, particularly at cell–cell junctions where it may act as an ion channel or transporter protein [8]. In fact, it was reported that STEAP1 may allow the transport of small molecules between adjacent cells, indicating that STEAP1 may be involved in intercellular communication [9,10]. Several studies have demonstrated the role of STEAP1 in cancer. In Ewing tumors, STEAP1 protein seems to promote cell growth and invasiveness by increasing intracellular reactive oxygen species levels. The oxidative stress that results from STEAP1 overexpression may enhance tumor aggressiveness through the activation of genes involved in cell proliferation and invasion [11]. Additionally, in gastric tumors, it was demonstrated that the upregulation of *STEAP1* increased cell proliferation, migration and invasion [12]. In human ovarian and lung cancers, the *STEAP1* gene is highly expressed, and it is associated with metastasis and epithelial–mesenchymal transition [12,13,14]. It was also demonstrated that the knockdown of *STEAP1* expression on prostate tumor cells is associated with antitumor effects, such as enhanced apoptosis, and reduced proliferation, migration and invasion [12,13,14,15]. Previously, it was shown that post-transcriptional and post-translational modifications may contribute to *STEAP1* overexpression, as *STEAP1* mRNA and protein stability are higher in neoplastic LNCaP cells than in non-neoplastic PNT1A cells [16]. However, these alterations do not justify the overexpression of *STEAP1* in tumor cells, suggesting that other mechanisms may be involved. Thus, we hypothesized that epigenetic alterations of the *STEAP1* gene result in its overexpression in PCa. The present study aimed to analyze the methylation pattern of the *STEAP1* gene in PCa cells. Therefore, we analyzed the methylation levels of the *STEAP1* gene promoter in prostate cell lines and human samples of PCa. In addition, we analyzed the association between methylation levels of *STEAP1* and gene expression using publicly available datasets. Additionally, non-neoplastic PNT1A cells were used to demonstrate that demethylation of the *STEAP1* gene, as well as a synergistic effect between DNA demethylation and inhibition of class I and II HDACs, induces *STEAP1* overexpression.

## 2. Materials and Methods

### 2.1. Prostate Cell Lines and Treatments

The human LNCaP PCa cell line and the immortalized non-neoplastic PNT1A prostate epithelial cell line were purchased from the European Collection of Cell Cultures (ECACC, Salisbury, UK). LNCaP and PNT1A cell lines were cultured at 37 °C in a 5% CO_2_ atmosphere with RPMI 1640 phenol-red medium (Sigma-Aldrich, St. Louis, MO, USA) supplemented with 10% fetal bovine serum (FBS, Sigma-Aldrich, USA) and 1% penicillin/streptomycin (Sigma-Aldrich, USA). For the treatment with the demethylation and the histone deacetylation drug (epidrugs), approximately 3 × 10^5^ PNT1A cells were seeded in six-well plates until reaching about 60% confluence. After that, PNT1A cells were exposed to one treatment with 5 µM 5-aza-dC (Sigma-Aldrich, USA) for 72 h, and the other with 5 µM 5-aza-dC for 48 h followed by 24 h with 1 µM TSA (Sigma-Aldrich, USA). In the control group, the medium was replaced by RPMI medium with DMSO for 72 h.

### 2.2. Patients and Tissue Sample Collection

Prostate tissue samples from five patients diagnosed with clinically localized PCa and primary treatment with radical prostatectomy, at the Portuguese Oncology Institute of Porto (IPO-Porto), were used in this study. Informed consent was obtained from all participants, according to institutional regulations. This study was approved by the institutional review board (Comissão de Ética para a Saúde-(IRB-CES-IPOFG-EPE 019/08)) of IPO-Porto.

### 2.3. DNA Extraction and Bisulfite Conversion

DNA extraction from PNT1A and LNCaP cells and clinical samples was carried out using the Gentra Puregene Cell Kit (Qiagen, Hilden, Germany) according to the manufacturer’s instructions. To evaluate the methylation pattern, 1 µg of genomic DNA was modified using the EZ DNA Methylation-Gold kit (ZYMO RESEARCH, Irvine, CA, USA) according to the manufacturer’s instructions. The modified DNA was stored at −80 °C.

### 2.4. Polymerase Chain Reaction (PCR) Amplification and Cloning Products

PCR reactions were performed using 200 ng of bisulfite-modified DNA in 25 μL reaction containing 1 U of TrueStart Hot Start Taq DNA Polymerase (Thermo Scientific, Waltham, MA, USA), 2.5 mM of MgCl_2_, 10 mM dNTPs and 300 nM of each primer (−338 fw/+74 rv). The primer sequences and characteristics are described in Table 1. The PCR products were purified using NucleoSpin Gel and the PCR Clean-up kit (Macherey-Nagel, Germany) according to the manufacturer’s instructions, cloned into a pNZY28 vector and transformed in NZYStar Competent Cells. After heat shock, the cells were plated onto LB agar plates containing 100 µg/mL ampicillin, 80 µg/mL X-gal and 0.5 mM IPTG and incubated at 37 °C overnight. All components used in cloning and transformation were purchased from Nzytech, Portugal.

### 2.5. DNA Sequencing

Colony screening was performed by PCR, with standard vector primers (T7 and M13), to confirm and amplify the DNA insert. Thus, white colonies were selected and incubated in 10 µL TE buffer at 100 °C for 2 min. Afterwards, 1 µL was used for PCR reaction with Speedy NZYTaq 2x Green Master Mix (Nzytech, Portugal). Sequencing of PCR products was carried out using the CEQ Dye Terminator Cycle Sequencing Quick Start Kit (Beckman Coulter, Fullerton, USA) according to the manufacturer’s instructions. For each sample, the DNA sequencing reaction was performed in both strands with universal sequencing primers (T7 and M13). The sequencing products were separated on an automated capillary DNA sequencer (GenomeLabTM GeXP, Genetic Analysis System; Beckman Coulter, Fullerton, CA, USA). The sequencing data analysis was performed using the Clustal Omega software to align the PCR product sequences with the sequence of *STEAP1* gene modified DNA.

### 2.6. Datasets and Bioinformatic Analysis

Three PCa datasets (GSE52955, GSE76938 and GSE38240) were downloaded from the public repository NCBI Gene Expression Omnibus (GEO) databases (https://www.ncbi.nlm.nih.gov/geo/, assessed on 12 November 2021). All these datasets were based on the GPL13534 platform (Illumina HumanMethylation450 BeadChip). For each dataset, only the samples associated with prostate were selected. Methylation status was determined through the interactive web tool GEO2R, which allows the comparison of the two groups defined, pathologic condition and normal. The methylation status varies between 0 and 1, where 0 means a low degree of methylation and 1 indicates a high degree of methylation. The results were exported, and graphs were constructed with GraphPad Prism 8.0.1. The details of each dataset used in the present study are described in Table 2. The correlation of DNA methylation status with *STEAP1* mRNA expression was assessed using another public repository, the Prostate Adenocarcinoma (TCGA, Cell 2015) [17] dataset, available from cBioPortal for Cancer Genomic (https://www.cbioportal.org/, assessed on 12 November 2021). This dataset comprises data from 333 primary prostate carcinomas.

### 2.7. Total RNA Extraction, cDNA Synthesis and Quantitative Real-Time PCR (qPCR)

Total RNA extraction from PNT1A cells treated with epidrugs (5-aza-dC and TSA) was carried out using TRI reagent (Sigma-Aldrich, USA) according to the manufacturer’s instructions. cDNA synthesis was performed using the NZY First-Strand cDNA Synthesis KIT (Nzytech, Portugal) according to the manufacturer’s instructions, and qPCR was carried out to evaluate the expression of *STEAP1* mRNA (h*STEAP1*) in PNT1A cells treated with 5-aza-dC alone and 5-aza-dC plus TSA. To normalize the expression of the *STEAP1* gene, human GAPDH (h*GAPDH*) and human beta-2-microglobulin (h*β2M*) primers were used as internal controls. qPCR reactions were carried out using 1 μL of cDNA synthesized in a 20 μL reaction containing 10 μL of Maxima SYBR Green/Fluorescein qPCR Master Mix (Thermo Scientific) and 300 nM of primer for each gene. Fold differences were calculated following the mathematical model proposed by Pfaffl using the formula: 2-(ΔΔCt) [21]. The primer sequence for each gene and respective amplicon sizes used in qPCR are described in Table 1.

### 2.8. Statistical Analysis

Data analysis was performed using GraphPad Prism version 8.0.1. for Windows (GraphPad Software, San Diego, CA, USA). The statistical significance of differences in *STEAP1* mRNA expression for the treatment with 5-aza-dC and TSA in non-neoplastic PNT1A cells was assessed by student’s *t*-test. Significant differences were considered when *p* < 0.05 compared to control values. All experimental data are shown as mean ± SEM.

## 3. Results

### 3.1. Methylation Analysis of STEAP1 Gene in Neoplastic Tissue/Cells Compared with Non-Neoplastic Cells

**Figure 1:**
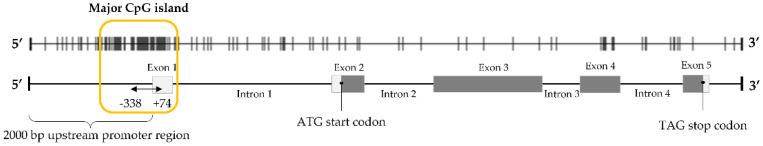
Schematic map of the predicted CpG island and indication of the region analyzed by bisulfite genomic sequencing within the exon–intron structure of *STEAP1* gene. Vertical bars represent the CG dinucleotides. Parameters used to find CpG islands: minimum length of island: 300 bp; maximum length of island: 2000 bp; C + Gs/total bases > 50%; CpG observed/CpG expected > 0.6.

To evaluate if DNA methylation plays a role in *STEAP1* gene regulation and if there are alterations in PCa, the methylation pattern of *STEAP1* was determined in PCa tissue samples, LNCaP and PNT1A cells. For this purpose, cytosine-rich regions of *STEAP1* gene promoter and primer design were performed using the Methyl Primer Express Software v1.0 (Applied Biosystems). This analysis indicated that part of the promoter region and the first exon of the *STEAP1* gene contain a large CpG island containing 24 CpG dinucleotides (Figure 1), which could provide a large number of sites for the methylation modification of this gene.

**Figure 2:**
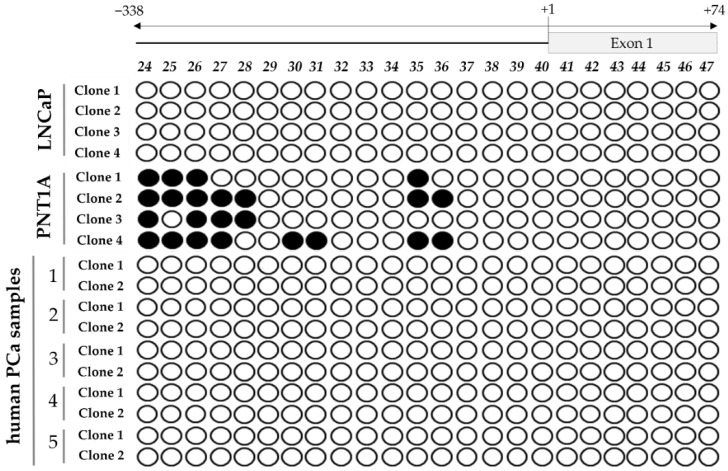
Evaluation of the *STEAP1* gene promoter methylation status in LNCaP and PNT1A cells, and human PCa samples. Each circle represents a CpG dinucleotide present in the CpG island identified, and on the top is the position of each CpG site relative to the beginning of the CpG island identified (● methylated CpG and ○ demethylated CpG). Each row represents a different clone.

The methylation pattern of the *STEAP1* was analyzed through the bisulfite sequencing PCR method from position −338 (promoter region) to +74 (exon 1). The analysis of the methylation pattern of *STEAP1* revealed some differences between neoplastic and non-neoplastic samples. In non-neoplastic PNT1A cells, the results show that some of the CpG dinucleotides located in the promoter region are methylated, but in PCa tissue or in neoplastic LNCaP cells the CpG dinucleotides are completely demethylated (Figure 2).

### 3.2. Analysis of STEAP1 Promoter Methylation Levels in PCa and Normal Prostate Tissues from the GEO Database

**Figure 3:**
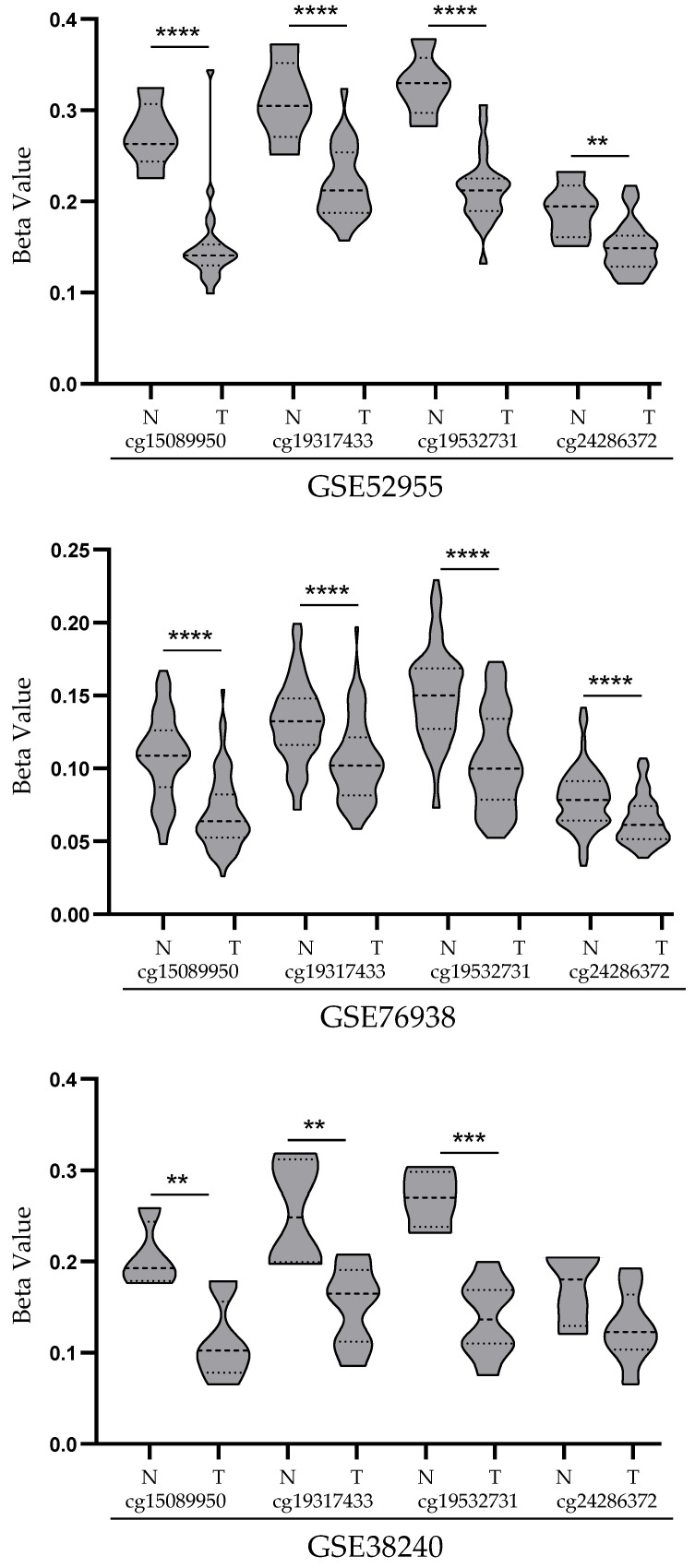
*STEAP1* promoter methylation levels (beta value) in GSE52955, GSE76938 and GSE38240 datasets. Each dataset represents methylation levels of *STEAP1* in prostate tumor tissue (T) and normal prostate tissue (N) for the four probes (cg15089950, cg19317433, cg19532731 and cg24286372). Violin plots were obtained with GraphPad Prism 8.0.1, and mean methylation levels between normal and tumor were compared with a student’s *t*-test. ** *p* < 0.01, *** *p* < 0.001 and **** *p* < 0.0001.

In order to support and validate the results above, the methylation pattern of *STEAP1* gene promoter methylation was evaluated in datasets from public PCa databases. The GSE52955, GSE76938 and GSE38240 datasets were downloaded and analyzed by GEO2R online software. Four CpG probes on the *STEAP1* gene promoter were selected: cg15089950, cg19317433, cg19532731 and cg24286372 located on −314 to −193 bp, −285 to −163 bp, −301 to −180 bp and −250 to −129 bp upstream of the transcription start, respectively. As shown in Figure 3, there were significant differences in the levels of CpG methylation between prostate tumor and normal tissue. In three datasets analyzed, the results reveal lower methylation levels of the *STEAP1* gene promoter in neoplastic tissue than in normal tissue.

### 3.3. Correlation between STEAP1 Gene Promoter Demethylation and Its Expression in PCa Tissue from the TCGA Database

**Figure 4:**
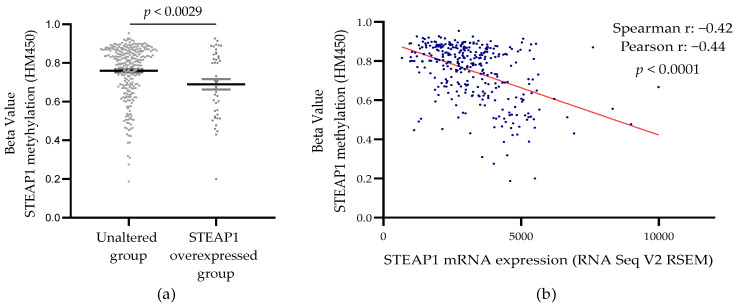
*STEAP1* methylation levels in PCa samples with normal and overexpression of *STEAP1* (**a**), and correlation between methylation levels and *STEAP1* mRNA expression (**b**) in Prostate Adenocarcinoma (TCGA, Cell 2015) [17] dataset (*n* = 333). Statistical analysis used a student’s *t*-test (**a**) and Spearman and Pearson correlation (**b**).

In an attempt to better understand whether DNA methylation status may have an impact on *STEAP1* gene expression, the Prostate Adenocarcinoma dataset was analyzed from The Cancer Genome Atlas (TCGA, Cell 2015) [17], accessed through the cBioPortal. In this dataset, differential methylation levels between the unaltered (*n* = 286) and altered (*n* = 43) expression of the *STEAP1* gene were observed (Figure 4a). Additionally, a negative correlation (Spearman coefficient of −0.42, and Pearson coefficient of −0.44) was observed between the methylation levels and the *STEAP1* gene expression (Figure 4b).

### 3.4. Effect of Epigenetic-Modulating Drugs in STEAP1 Gene Expression in Non-Neoplastic PNT1A Cells

**Figure 5:**
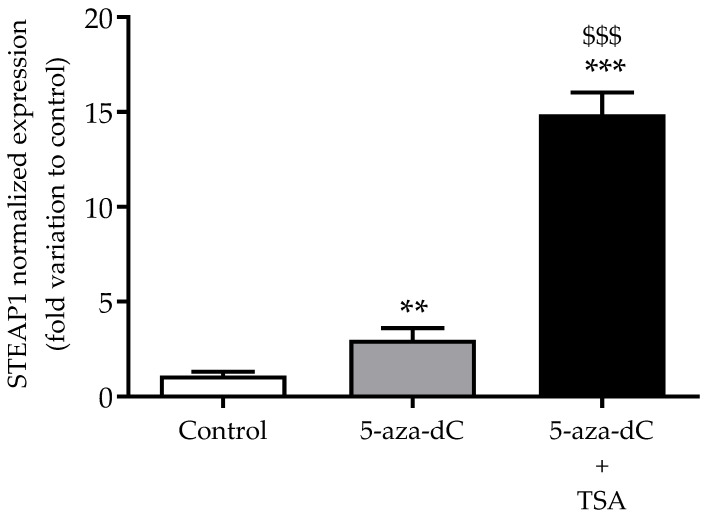
Effect of treatment with 5-aza-dC and TSA (DNMT and HDAC inhibitors, respectively) on *STEAP1* mRNA expression in PNT1A cells. Relative *STEAP1* mRNA expression was determined by qPCR analysis after normalization with the GAPDH and β2M housekeeping genes. Results are expressed as fold-variation relative to the control group. Error bars indicate mean ± SEM (*n* = 6). ** *p* < 0.01 and *** *p* < 0.001 relative to control. ^$$$^ *p* < 0.001 relative to 5-aza-dC.

In order to support that epigenetic modifications contribute to the regulation of *STEAP1* expression, non-neoplastic PNT1A cells were used to evaluate the effect of DNMT and HDAC inhibitors (5-aza-dC and TSA, respectively) on *STEAP1* mRNA expression by qPCR. As shown in Figure 5, treatment with the demethylation agent 5-aza-dC induced a three-fold increase in *STEAP1* mRNA levels when compared to the control group (*p* < 0.01). Moreover, treatment with both epidrugs (5-aza-dC + TSA), which contributes to demethylation and histone hyperacetylation, induced a strong increase (15-fold variation relative to control, *p* < 0.001) in *STEAP1* mRNA levels.

### 3.5. Analysis of Co-Expression between STEAP1 mRNA Expression and HDACs

The TSA drug is a potent and specific inhibitor of HDAC classes I and II, which include ten isoforms (HDAC1–HDAC10) [22]. Considering that the results above suggest a negative association between histone deacetylation and *STEAP1* mRNA expression, we intended to analyze the association between *STEAP1* mRNA expression and *HDAC* mRNA expression, using the Prostate Adenocarcinoma (TCGA, Cell 2015) [17] dataset. Of the ten isoforms analyzed, *STEAP1* mRNA expression showed a positive association with *HDAC8*, as highlighted in Table 3. On the other hand, *STEAP1* mRNA expression was negatively associated with *HDAC4,5,7* and *9* (Table 3).

## 4. Discussion

The methylation pattern observed in normal tissues undergoes relevant modifications in cancer, leading to changes in the regulation of the transcription of numerous genes [23]. Recent studies have shown that *STEAP1* acts as an oncogene, showing that its overexpression in several human cancers contributes to tumor progression and aggressiveness through the inhibition of apoptosis and stimulation of cell proliferation, invasion and epithelial–mesenchymal transition [11,12,13,14,15]. Although the stability of the *STEAP1* gene and protein is higher in LNCaP PCa cells than in PNT1A cells, contributing to STEAP1 overexpression, other mechanisms underlying its overexpression in cancer must be involved. As epigenetics has been pointed out as a major hallmark in cancer, affecting genes involved in all cellular pathways [24,25], our main goal was to assess whether epigenetic mechanisms are involved in the regulation of the *STEAP1* gene expression in PCa, and if there are changes between normal and PCa cells.

As a first approach, two different cell lines were chosen, neoplastic LNCaP and non-neoplastic PNT1A, which express high and low levels of STEAP1, respectively [16], to analyze the methylation status of the *STEAP1* gene. Furthermore, five human PCa samples were also analyzed. The major CpG island located from position −338 of the promoter region to position +74 of the first exon of *STEAP1* revealed differences in the methylation status. While in non-neoplastic PNT1A cells, the CpG dinucleotides near the transcription start site are methylated, in neoplastic LNCaP cells and PCa samples these are demethylated. This result suggests that demethylation of the *STEAP1* gene promoter may lead to its overexpression in PCa. Hypomethylation/demethylation-dependent overexpression of several oncogenes has already been described in several cancer types. In PCa cells, Wingless-related MMTV integration site 5A, S100 calcium-binding protein P and cysteine-rich protein 1 were found to be hypomethylated [26]. hsa-miR-191 was also hypomethylated in 63% of hepatocellular carcinoma tissue samples, associated with its increased expression [27], and demethylation of the miR-128a promoter region drives the upregulation of miR-128a expression in the human T lymphocyte Jurkat cell line [28].

The possibility that demethylation of the *STEAP1* gene promoter may be involved in its overexpression in PCa was also corroborated by a bioinformatic analysis, using public datasets from the GEO database and cBioPortal platform. This analysis showed that CpG dinucleotides in the *STEAP1* gene of PCa samples have low levels of methylation, negatively correlated with *STEAP1* mRNA expression. These results suggest, once more, that demethylation of the *STEAP1* gene promoter may contribute to its overexpression in PCa. Our results are in line with other studies that also analyzed genes of the STEAP family, which showed that epigenetic alterations may be responsible for changes in gene expression. Using combined analysis of GEO and TCGA datasets, it was shown that *STEAP3* is hypomethylated and consequently upregulated in glioblastoma, and may be used as a potential methylation-based prognostic biomarker. In addition, the authors suggested that *STEAP3* is a potential target for glioblastoma treatment [29]. Another gene that was also reported to be deregulated due to epigenetic changes is the *STEAP4* gene. Tamura et al. showed no CpG methylation in the *STEAP4* promoter region in LNCaP cells, suggesting that demethylation may activate the expression of the *STEAP4* gene in PCa cells [30]. On the other hand, a more recent study showed that *STEAP4* was hypermethylated and downregulated in the hepatocellular carcinoma when compared to the non-tumor liver tissues [31]. This study also demonstrated that expression of *STEAP4* was restored with a DNA methyltransferase (DNMTs) inhibitor and a histone deacetylase (HDACs) inhibitor, suggesting that aberrant DNA methylation suppressed the expression of the *STEAP4* gene [31].

It is well established that there is a crosstalk between DNA methylation and histone modifications in the control of gene expression [32,33]. Aberrant CpG-island methylation by recruiting DNMTs and HDACs might be directly targeted by consequence of gene expression alterations [34]. Therefore, it was hypothesized that treatment with both DNMT and HDAC inhibitors might enhance *STEAP1* expression in cells with reduced levels of STEAP1. Thus, the non-neoplastic PNT1A cell line was used to evaluate the effects of 5-aza-dC (DNMT inhibitor) plus TSA (HDAC inhibitor) on the expression of STEAP1. The results indicate an increase in *STEAP1* gene expression in response to treatment with DNMT and HDAC inhibitors, suggesting a synergistic effect of combined hypomethylation and histone hyperacetylation.

HDACs are enzymes that remove acetyl groups from the tails of histones, resulting in a more closed chromatin structure and repression of gene expression [22,35,36]. Class I of the HDAC family includes HDAC1, 2, 3 and 8; class II includes HDAC4, 5, 6, 7, 9 and 10 [36]. To the best of our knowledge, the role played by both HDAC class I and II enzymes in the regulation of *STEAP1* gene expression is still unknown. So, we aimed to explore the association between HDAC and *STEAP1* gene expression. Our results reveal a positive association of *HDAC8* and a negative association of *HDAC4,5,7* and *9* with *STEAP1* mRNA expression. In fact, the HDAC family modulates several genes involved in cancer development/progression via angiogenesis, cell adhesion, migration and invasion [22]. Some studies support our results, showing that increased expression of autotaxin in PCa cell lines is mediated by the downregulation of *HDAC7* and *HDAC3* [37]; additionally, *HDAC5* is downregulated in human cancer, namely PCa [38], and decreased expression of *HDAC4* increases the growth of PCa cells [39]. On the other hand, there are also studies showing an overexpression of HDAC in several types of cancer, suggesting the use of HDAC inhibitors as a promising class of compounds for cancer treatment [40,41,42,43]. Thus, the effects that can be triggered by these inhibitors on oncogenes should not be ignored and more studies are required to clarify their effectiveness in cancer treatment.

To summarize, this study showed that the *STEAP1* gene is hypomethylated in PCa cells when compared to non-neoplastic cells, contributing to the overexpression of *STEAP1* in PCa. Furthermore, our results suggest a putative involvement of *HDCA4,5,7* and *9* in the regulation of *STEAP1* in PCa. Considering the complexity of the mechanisms associated with HDAC, more studies are required to clarify their role in *STEAP1* regulation, as well as to elucidate this association with PCa development and progression.

## Figures and Tables

**Table 1:** Primer sequences and respective amplicon size used for amplification of the modified DNA from cell lines or human samples, and for the quantitative real-time PCR.

Primers	Accession Number	Sequence	Amplicon Size (bp)	Target
*STEAP1*_−338 fw *STEAP1*_+74 rv	NC_000007.14	5′ AAAGTGTGATTTGGGAATGTTTTT 3′ 5′ TTTTAAGTTAGTTGTAGGTTTT 3′	412	Modified DNA
h*STEAP1*_619 fw h*STEAP1*_747 rv	NM_012449.3	5′ GGCGATCCTACAGATACAAGTTGC 3′ 5′ CCAATCCCACAATTCCCAGAGAC 3′	128	mRNA
h*GAPDH*_74 fw h*GAPDH*_149 rv	NM_002046.7	5′ CGCCAGCCGAGCCACATC 3′ 5′ CGC CCA ATA CGA CCA AAT CCG 3′	75	mRNA
h*β2M*_347 fw h*β2M*_439 rv	NM_004048.4	5′ ATGAGTATGCCTGCCGTGTG 3′ 5′ CAAACCTCCATGATGCTGCTTAC 3′	92	mRNA

**Table 2:** Dataset used to evaluate the *STEAP1* gene methylation profiling.

Dataset	Platform	Sample Type	Disease Condition (*n*)	Normal Tissue (*n*)	Reference
GSE52955	GLP13534	Frozen tissue	Cancer (25)	5 *	[18]
GSE76938	GLP13534		Cancer (73)	Adjacent Tissue (63)	[19]
GSE38240	GLP13534		Cancer Metastasis (8)	4 ^#^	[20]

* Obtained from patients submitted to cystoprostatectomy due to bladder cancer. ^#^ Obtained from organ donor with no evidence of prostate cancer.

**Table 3:** Association of *STEAP1* mRNA expression with class I and II HDACs, showing spearman’s rank for each comparison and the respective *p*-value. The associations with significant values are highlighted in bold.

*STEAP1* Correlated	Spearman’s Correlation	*p*-Value
HDAC1	−0.011	0.834
HDAC2	−0.005	0.929
HDAC3	0.089	0.105
**HDAC4**	**−0.242**	**8.07 × 10^−6^**
**HDAC5**	**−0.305**	**1.35 × 10^−8^**
HDAC6	0.049	0.366
**HDAC7**	**−0.336**	**3.31 × 10^−10^**
**HDAC8**	**0.255**	**2.34 × 10^−6^**
**HDAC9**	**−0.294**	**4.53 × 10^−8^**
HDAC10	−0.064	0.245

## References

[1] Culp M.B.B., Soerjomataram I., Efstathiou J.A., Bray F., Jemal A. (2020). Recent Global Patterns in Prostate Cancer Incidence and Mor-tality Rates. European Urology.

[2] Gonzalgo M.L., Isaacs W.B. (2003). Molecular Pathways to Prostate Cancer. J. Urol..

[3] Reynolds M.A. (2008). Molecular alterations in prostate cancer. Cancer Lett..

[4] Joshua A.M., Evans A., Van der Kwast T., Zielenska M., Meeker A.K., Chinnaiyan A., Squire J.A. (2008). Prostatic preneoplasia and beyond. Biochim. Biophys. Acta..

[5] Nelson W.G., De Marzo A.M., Yegnasubramanian S. (2009). Epigenetic alterations in human prostate cancers. Endocrinology.

[6] Wang R., Liu X. (2019). Epigenetic regulation of prostate cancer. Genes Dis..

[7] Kulis M., Esteller M. (2010). DNA methylation and cancer. Adv. Genet..

[8] Hubert R.S., Vivanco I., Chen E., Rastegar S., Leong K., Mitchell S.C., Madraswala R., Zhou Y., Kuo J., Raitano A.B. (1999). STEAP: A prostate-specific cell-surface antigen highly expressed in human prostate tumors. Proc. Natl. Acad. Sci. USA.

[9] Yamamoto T., Tamura Y., Kobayashi J.-I., Kamiguchi K., Hirohashi Y., Miyazaki A., Torigoe T., Asanuma H., Hiratsuka H., Sato N. (2013). Six-transmembrane epithelial antigen of the prostate-1 plays a role for in vivo tumor growth via intercellular communication. Exp. Cell Res..

[10] Challita-Eid P.M., Morrison K., Etessami S., An Z., Morrison K.J., Perez-Villar J.J., Raitano A.B., Jia X.C., Gudas J.M., Kanner S.B. (2007). Monoclonal antibodies to six-transmembrane epithelial antigen of the prostate-1 inhibit intercellular communication in vitro and growth of human tumor xenografts in vivo. Cancer Res..

[11] Grunewald T.G.P., Diebold I., Esposito I., Plehm S., Hauer K., Thiel U., Da Silva-Buttkus P., Neff F., Unland R., Müller-Tidow C. (2012). STEAP1 is associated with the invasive and oxidative stress phenotype of ewing tumors. Mol. Cancer Res..

[12] Zhang Z., Hou W., Zhang C., Tan Y., Zhang D., An W., Pan S., Wu W., Chen Q., Xu H. (2020). A research of STEAP1 regulated gastric cancer cell proliferation, migration and invasion in vitro and in vivos. J. Cell. Mol. Med..

[13] Huo S.-F., Shang W.-L., Yu M., Ren X.-P., Wen H.-X., Chai C.-Y., Sun L., Hui K., Liu L.-H., Wei S.-H. (2020). STEAP1 facilitates metastasis and epithelial-mesenchymal transition of lung adenocarcinoma via the JAK2/STAT3 signaling pathway. Biosci. Rep..

[14] Jiao Z., Huang L., Sun J., Xie J., Wang T., Yin X., Zhang H., Chen J. (2020). Six-transmembrane epithelial antigen of the prostate 1 ex-pression promotes ovarian cancer metastasis by aiding progression of epithelial-to-mesenchymal transition. Histochem. Cell Biol..

[15] Gomes I.M., Rocha S., Gaspar C., Alvelos M.I., Santos C.R., Socorro S., Maia C.J. (2018). Knockdown of STEAP1 inhibits cell growth and induces apoptosis in LNCaP prostate cancer cells counteracting the effect of androgens. Med. Oncol..

[16] Gomes I.M., Santos C.R., Maia C.J. (2014). Expression of STEAP1 and STEAP1B in prostate cell lines, and the putative regulation of STEAP1 by post-transcriptional and post-translational mechanisms. Genes Cancer.

[17] The Cancer Genome Atlas Research Network (2015). The Molecular Taxonomy of Primary Prostate Cancer. Cell.

[18] Ramalho-Carvalho J., Gonçalves C., Graça I., Bidarra D., Pereira-Silva E., Salta S., Godinho M.I., Gomez A., Esteller M., Costa B. (2018). A multiplatform approach identifies miR-152-3p as a common epigenetically regulated onco-suppressor in prostate cancer targeting TMEM97. Clin. Epigenetics.

[19] Kirby M.K., Ramaker R.C., Roberts B.S., Lasseigne B.N., Gunther D.S., Burwell T.C., Davis N.S., Gulzar Z.G., Absher D.M., Cooper S.J. (2017). Genome-wide DNA methylation measurements in prostate tissues uncovers novel prostate cancer diagnostic biomarkers and transcription factor binding patterns. BMC Cancer.

[20] Aryee M.J., Liu W., Engelmann J.C., Nuhn P., Gurel M., Haffner M.C., Esopi D., Irizarry R.A., Getzenberg R.H., Nelson W.G. (2013). DNA methylation alterations exhibit intra-individual stability and inter-individual heterogeneity in prostate cancer metastases. Sci. Transl. Med..

[21] Pfaffl M.W. (2001). A new mathematical model for relative quantification in real-time RT-PCR. Nucleic Acids Res..

[22] Abbas A., Gupta S. (2008). The role of histone deacetylases in prostate cancer. Epigenetics.

[23] Sharma S., Kelly T.K., Jones P.A. (2010). Epigenetics in cancer. Carcinogenesis.

[24] Kukkonen K., Taavitsainen S., Huhtala L., Uusi-Makela J., Granberg K., Nykter M., Urbanucci A. (2021). Chromatin and Epigenetic Dysregulation of Prostate Cancer Development, Progression, and Therapeutic Response. Cancers.

[25] Darwiche N. (2020). Epigenetic mechanisms and the hallmarks of cancer: An intimate affair. Am. J. Cancer Res..

[26] Wang Q., Williamson M., Bott S., Brookman-Amissah N., Freeman A., Nariculam J., Hubank M., Ahmed A., Masters J.R. (2007). Hypomethylation of WNT5A, CRIP1 and S100P in prostate cancer. Oncogene.

[27] He Y., Cui Y., Wang W., Gu J., Guo S., Ma K., Luo X. (2011). Hypomethylation of the hsa-miR-191 locus causes high expression of hsa-mir-191 and promotes the epithelial-to-mesenchymal transition in hepatocellular carcinoma. Neoplasia.

[28] Yamada N., Noguchi S., Kumazaki M., Shinohara H., Miki K., Naoe T., Akao Y. (2014). Epigenetic regulation of microRNA-128a ex-pression contributes to the apoptosis-resistance of human T-cell leukaemia jurkat cells by modulating expression of fas-associated protein with death domain (FADD). Biochim. Biophys. Acta..

[29] Zhang M., Lv X., Jiang Y., Li G., Qiao Q. (2019). Identification of aberrantly methylated differentially expressed genes in glioblastoma multiforme and their association with patient survival. Exp. Ther. Med..

[30] Tamura T., Chiba J. (2009). STEAP4 regulates focal adhesion kinase activation and CpG motifs within STEAP4 promoter region are frequently methylatedin DU145, human androgen-independent prostate cancer cells. Int. J. Mol. Med..

[31] Yamada N., Yasui K., Dohi O., Gen Y., Tomie A., Kitaichi T., Iwai N., Mitsuyoshi H., Sumida Y., Moriguchi M. (2016). Genome-wide DNA methylation analysis in hepatocellular carcinoma. Oncol. Rep..

[32] Esteller M. (2007). Cancer epigenomics: DNA methylomes and histone-modification maps. Nat. Rev. Genet..

[33] Di Croce L., Raker V.A., Corsaro M., Fazi F., Fanelli M., Faretta M., Fuks F., Lo Coco F., Kouzarides T., Nervi C. (2002). Methyltransferase recruitment and DNA hypermethylation of target promoters by an oncogenic transcription factor. Science.

[34] D’Alessio A.C., Szyf M. (2006). Epigenetic tête-à-tête: The bilateral relationship between chromatin modifications and DNA methylation. Biochem. Cell Biol..

[35] Minucci S., Pelicci P.G. (2006). Histone deacetylase inhibitors and the promise of epigenetic (and more) treatments for cancer. Nat. Rev. Cancer.

[36] Park S.-Y., Kim J.-S. (2020). A short guide to histone deacetylases including recent progress on class II enzymes. Exp. Mol. Med..

[37] Li S., Wang B., Xu Y., Zhang J. (2011). Autotaxin is induced by TSA through HDAC3 and HDAC7 inhibition and antagonizes the TSA-induced cell apoptosis. Mol. Cancer.

[38] Zhou Y., Jin X., Ma J., Ding D., Huang Z., Sheng H., Yan Y., Pan Y., Wei T., Wang L. (2021). HDAC5 Loss Impairs RB Repression of Pro-Oncogenic Genes and Confers CDK4/6 Inhibitor Resistance in Cancer. Cancer Res..

[39] Yang Y., Tse A.K.-W., Li P., Ma Q., Xiang S., Nicosia S.V., Seto E., Zhang X., Bai W. (2011). Inhibition of androgen receptor activity by histone deacetylase 4 through receptor SUMOylation. Oncogene.

[40] Hontecillas-Prieto L., Flores-Campos R., Silver A., De Álava E., Hajji N., García-Domínguez D.J. (2020). Synergistic Enhancement of Cancer Therapy Using HDAC Inhibitors: Opportunity for Clinical Trials. Front. Genet..

[41] Pacheco M.B., Camilo V., Lopes N., Moreira-Silva F., Correia M.P., Henrique R., Jerónimo C. (2021). Hydralazine and Panobinostat Attenuate Malignant Properties of Prostate Cancer Cell Lines. Pharmaceuticals.

[42] Lakshmaiah K.C., Jacob L.A., Aparna S., Lokanatha D., Saldanha S.C. (2014). Epigenetic therapy of cancer with histone deacetylase in-hibitors. J. Cancer Res. Ther..

[43] Rana Z., Diermeier S., Hanif M., Rosengren R.J. (2020). Understanding Failure and Improving Treatment Using HDAC Inhibitors for Prostate Cancer. Biomedicines.

